# Distinct µ-opioid ensembles trigger positive and negative fentanyl reinforcement

**DOI:** 10.1038/s41586-024-07440-x

**Published:** 2024-05-22

**Authors:** Fabrice Chaudun, Laurena Python, Yu Liu, Agnes Hiver, Jennifer Cand, Brigitte L. Kieffer, Emmanuel Valjent, Christian Lüscher

**Affiliations:** 1 Department of Basic Neurosciences, Faculty of Medicine, University of Geneva, Geneva, Switzerland; 2 INSERM U1114, University of Strasbourg Institute for Advanced Study, Strasbourg, France; 3 IGF, Université de Montpellier CNRS, Inserm, Montpellier, France; 4 Clinic of Neurology, Department of Clinical Neurosciences, Geneva University Hospital, Geneva, Switzerland

**Keywords:** Cellular neuroscience, Diseases of the nervous system

## Abstract

Fentanyl is a powerful painkiller that elicits euphoria and positive reinforcement^1^. Fentanyl also leads to dependence, defined by the aversive withdrawal syndrome, which fuels negative reinforcement^2,3^ (that is, individuals retake the drug to avoid withdrawal). Positive and negative reinforcement maintain opioid consumption, which leads to addiction in one-fourth of users, the largest fraction for all addictive drugs^4^. Among the opioid receptors, µ-opioid receptors have a key role^5^, yet the induction loci of circuit adaptations that eventually lead to addiction remain unknown. Here we injected mice with fentanyl to acutely inhibit γ-aminobutyric acid-expressing neurons in the ventral tegmental area (VTA), causing disinhibition of dopamine neurons, which eventually increased dopamine in the nucleus accumbens. Knockdown of µ-opioid receptors in VTA abolished dopamine transients and positive reinforcement, but withdrawal remained unchanged. We identified neurons expressing µ-opioid receptors in the central amygdala (CeA) whose activity was enhanced during withdrawal. Knockdown of µ-opioid receptors in CeA eliminated aversive symptoms, suggesting that they mediate negative reinforcement. Thus, optogenetic stimulation caused place aversion, and mice readily learned to press a lever to pause optogenetic stimulation of CeA neurons that express µ-opioid receptors. Our study parses the neuronal populations that trigger positive and negative reinforcement in VTA and CeA, respectively. We lay out the circuit organization to develop interventions for reducing fentanyl addiction and facilitating rehabilitation.

## Abstract (web summary)

Experiments using fentanyl treatment of mice show that µ-opioid receptors mediate positive reinforcement in the ventral tegmental area and negative reinforcement in central amygdala, thereby identifying the circuits that lead to opioid addiction.

## Main

Fentanyl addiction is a pressing public health concern and is causing increasing numbers of overdoses and high addiction rates. Fentanyl is particularly addictive because of its potency and rapid kinetics, causing strong euphoria and behavioural reinforcement. A highly aversive withdrawal syndrome manifests upon abrupt termination of fentanyl exposure^3,6^. Consequently, individuals with fentanyl addiction develop elaborate strategies to avoid withdrawal, a behaviour that reflects negative reinforcement^7^. Fentanyl addiction is thus the result of positive and negative reinforcement converging on circuits that govern the transition from controlled to compulsive consumption. Early studies in rats have identified that withdrawal expression is widespread throughout the brain^8^, leading to the notion that distinct neural circuits drive specific withdrawal symptoms. Additionally, brain-wide genetic deletion of the µ-opioid receptors (µORs) prevents the induction of positive and negative reinforcement, as both conditioned place preference (CPP) and withdrawal are abolished in these mice^5^. Although the neurons at the origin of positive reinforcement are believed to reside in VTA^9–13^, whether the same neural population also mediates negative reinforcement remains unknown.

GABA neurons (γ-aminobutyric acid-expressing neurons) in VTA express µORs that rapidly inhibit these cells via G_io_ proteins^14,15^. Long-lasting receptor activation can cause signalling adaptations such as cAMP supersensitization and increased cellular activity upon signalling termination^16^. An appealing hypothesis therefore suggests that the symptoms of withdrawal stem from overactivity of VTA GABA neurons. Upon termination of the opioid exposure, these adaptations would result in suppression of dopamine neuron activity, lowering accumbal dopamine levels and thus causing dysphoria^17^. To test this hypothesis, we used a combination of behavioural assays, in vivo recordings and genetic manipulations to disentangle the underlying neural circuitry responsible for the dual reinforcement.

## Neural circuits that induce reinforcements

**Fig. 1:**
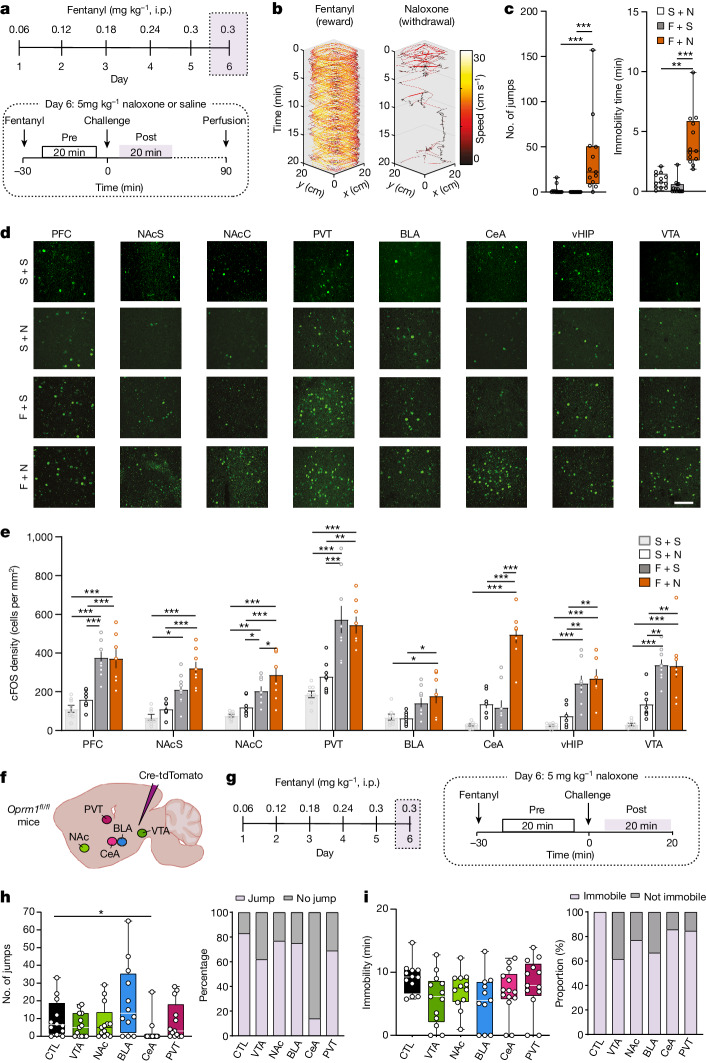
Cellular determinant of fentanyl reward and aversion. **a**, Experimental schedule. i.p., intraperitoneal injection. **b**, Representative example of speed dynamics following intraperitoneal injection of fentanyl and during precipitation of withdrawal by naloxone. **c**, Box plot of jumps and immobility time in dependent mice without precipitation (grey, *n* = 14), in dependent mice with precipitation (red, *n* = 13) and in non-dependent mice with naloxone injection (white, *n* = 13; jump Kruskal–Wallis test: *H*(3) = 26.79, *P* < 0.001; immobility time Kruskal–Wallis test *H*(3) = 28.94, *P* < 0.001; Dunn’s multiple comparisons test, **P < 0.01, ***P < 0.001). F, fentanyl; N, naloxone; S, saline. **d**, Representative images of cFOS staining in prefrontal cortex (PFC), NAc shell (NAcS), NAc core (NAcC), paraventricular thalamus (PVT), basolateral amygdala (BLA), CeA, ventral hippocampus (vHIP) and VTA in a non-dependent mouse after saline injection (top row), naloxone injection (second row), dependent mice without precipitated withdrawal (third row) and dependent mice with precipitated withdrawal (bottom row). Scale bar, 100 μm. **e**, Number of cFOS-positive cells in the brain areas shown in **d** (*n* = 7–9 mice per group, two-way ANOVA; PFC: fentanyl effect *F*_(1,28)_ = 59.68, *P* < 0.001, naloxone effect *F*_(1,28)_ = 0.50, *P* > 0.05, interaction *F*_(1,28)_ = 0.67, *P* > 0.05; NAcS: fentanyl effect *F*_(1,28)_ = 30.84, *P* < 0.001, naloxone effect *F*_(1,28)_ = 5.64, *P* < 0.05, interaction *F*_(1,28)_ = 0.59, *P* > 0.05; NAcC: fentanyl effect *F*_(1,29)_ = 55.43, *P* < 0.001, naloxone effect *F*_(1,29)_ = 10.34, *P* < 0.01, interaction *F*_(1,29)_ = 2.13, *P* > 0.05; PVT: fentanyl effect *F*_(1,29)_ = 48.72, *P* < 0.001, naloxone effect *F*_(1,28)_ = 0.47, *P* > 0.05, interaction *F*_(1,29)_ = 1.65, *P* > 0.05; BLA: fentanyl effect *F*_(1,29)_ = 13.73, *P* < 0.001, naloxone effect *F*_(1,29)_ = 0.39, *P* > 0.05, interaction *F*_(1,29)_ = 0.73, *P* > 0.05; CeA: fentanyl effect *F*_(1,29)_ = 51.86, *P* < 0.001, naloxone effect *F*_(1,29)_ = 60.56, *P* < 0.001, interaction *F*_(1,29)_ = 18.54, *P* < 0.001; vHYP: fentanyl effect *F*_(1,25)_ = 42.72, *P* < 0.001, naloxone effect *F*_(1,25)_ = 1.37, *P* > 0.05, interaction *F*_(1,25)_ = 0.13, *P* > 0.05; VTA: fentanyl effect *F*_(1,29)_ = 51.48, *P* < 0.001, naloxone effect *F*_(1,29)_ = 2.00, *P* > 0.05, interaction *F*_(1,29)_ = 2.44, *P* > 0.05, Bonferoni’s multiple comparisons test. *P < 0.05, **P < 0.01, ***P < 0.001). Data are mean ± s.e.m. **f**, Schematic of mouse preparation to induce µOR knockdown in various brain regions (control (CTL), *n* = 12; VTA, *n* = 13; NAc, *n* = 13; BLA, *n* = 12; CeA, *n* = 14; PVT, *n* = 13). **g**, Left, schedule of experiment to induce fentanyl dependence. Right, behavioural test to evaluate precipitated withdrawal induced by intraperitoneal injection of 5 mg kg^−1^ naloxone. **h**,**i**, Left, box plot of precipitated jump (**h**) and immobility (**i**) withdrawal symptoms after µOR deletion in indicated brain areas (CTL, *n* = 12; VTA, *n* = 13; NAc, *n* = 13; BLA, *n* = 12; CeA, *n* = 14; PVT, *n* = 13). Right, proportion of mice showing the presence of at least one precipitated jump (**h**) and at least 5 min of immobility (**i**) withdrawal symptoms. Kruskal–Wallis test: jumps, *H*(6) = 15.39, *P* < 0.01; immobility, *H*(6) = 8.774, *P* = 0.12; Dunn’s multiple comparisons test, *P = 0.0218. In box plots, the centre line is the median, box edges delineate first and third quartiles, and whiskers extend to maximum and minimum values. Source Data

**Extended Data Fig. 1:**
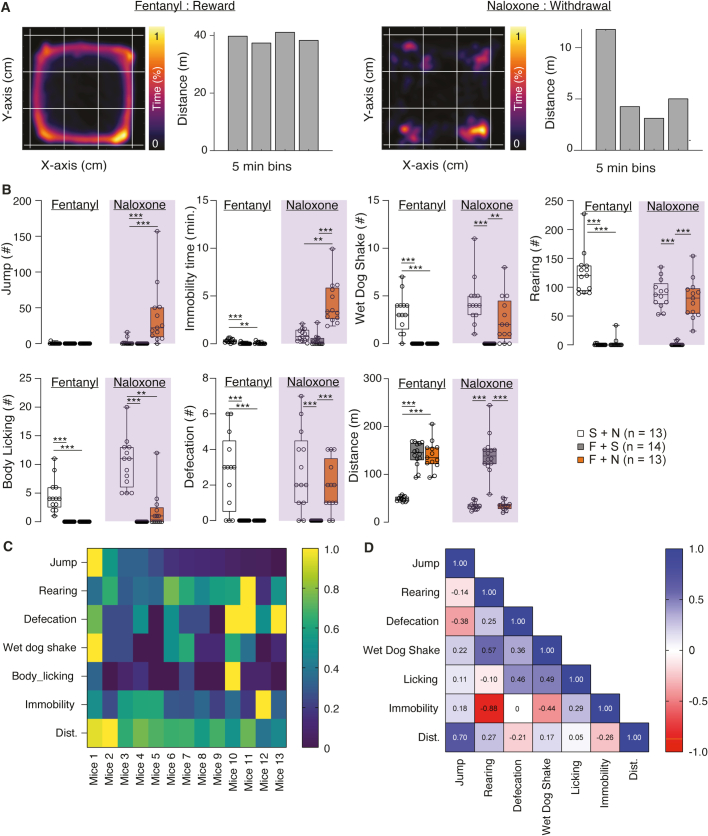
Behavioral quantification of fentanyl precipitated withdrawal. (**A**) Representative example of occupancy (left) and distance traveled (Right) after i.p. injection of fentanyl (Reward, left panel) or during precipitation of withdrawal by an i.p. injection of naloxone (Withdrawal, right panel). (**B**) Box Plot representation (Quartils and median, whiskers min to max) of the seven withdrawal symptoms quantified in dependent animal with no precipitation (grey, *n* = 14), in dependent animal with precipitation (red, *n* = 13) and in non-dependent animal with naloxone injection (white, *n* = 13). (During fentanyl: Kruskal-Wallis test: Rearing, H(3) = 31.72, P < 0.001, Jump, H(3) = 2.077, Defecation, H(3) = 26.32, P < 0.001, Wet Dog Shake, H(3) = 33.37, P < 0.001, Body Licking, H(3) = 37.14, P < 0.001, Immobility Time, H(3) = 20.47, P < 0.001, Distance, H(3) = 25.84, P < 0.001; During Naloxone: Kruskal-Wallis test: Rearing, H(3) = 27.67, Jump, H(3) = 26.79, P < 0.001, Defecation, H(3) = 21.65, P < 0.001, Wet Dog Shake, H(3) = 25.83, P < 0.001, Body Licking, H(3) = 30.14, P < 0.001, Immobility Time, H(3) = 28.94, P < 0.001, Distance, H(3) = 26.70; P < 0.001; Dunn’s multiple comparisons test, *p < 0.05, **p < 0.01, ***p < 0.001). (**C**) Heat-map representation of the different precipitated withdrawal symptoms normalized individually from min to max (respectively 0 and 1). (**D**) Correlation matrix of the individual precipitated withdrawal symptoms. Superimposed are annotated the Spearman correlation coefficient. Source Data

We first injected mice with an increasing daily dose of fentanyl (0.06, 0.12, 0.18, 0.24 and 0.30 mg kg, intraperitoneal) for 5 days, and then precipitated withdrawal with the opioid antagonist naloxone (5 mg kg^−1^; Fig. 1a). Naloxone readily terminated fentanyl-induced locomotion (Fig. 1b and Extended Data Fig. 1a), leading to increased immobility only interrupted by jumps (Fig. 1c). Rearing, defecation, body licking and wet-dog shakes were elicited even in fentanyl-naive mice but could be controlled by an additional injection on day 6 (Extended Data Fig. 1b), suggesting an endogenous opioid tone. We observed no interactions between the different symptoms (Extended Data Fig. 1c,d), confirming that the withdrawal syndrome comprises peripheral (for example, diarrhea) and central symptoms that are largely independent. Given the selective effect of naloxone for jumps and immobility in fentanyl-exposed mice, we focused on these symptoms.

**Extended Data Fig. 2:**
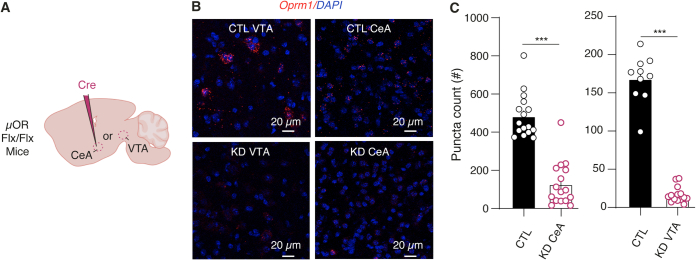
Anatomical validation of the KD experiment. (**A**) Schematic of the mice preparation to induce VTA µORs deletion. (**B**) Fluorescent in situ hybridization (RNAscope) for *Oprm1* (µORs, red) and DAPI (blue) in VTA (Left) or CeA (Right) without (Top) or with µORs deletion (Bottom) (**C**) Quantification of mRNA puncta (Mean ± SEM) per images in control (black) vs KD animals (red) respectively in VTA (Left) and CeA (Right). For VTA, *n* (images) = 14 and *n* (images) = 10, respectively for KD animals (*n* = 3 mice) and control animals (*n* = 3 mice). For CeA, *n* (images) = 16 and *n* (images) = 16, (*n* = 7 mice) (unilateral injection). Mann-Whitney test, two-sided, ***p < 0.001. Source Data

**Extended Data Fig. 3:**
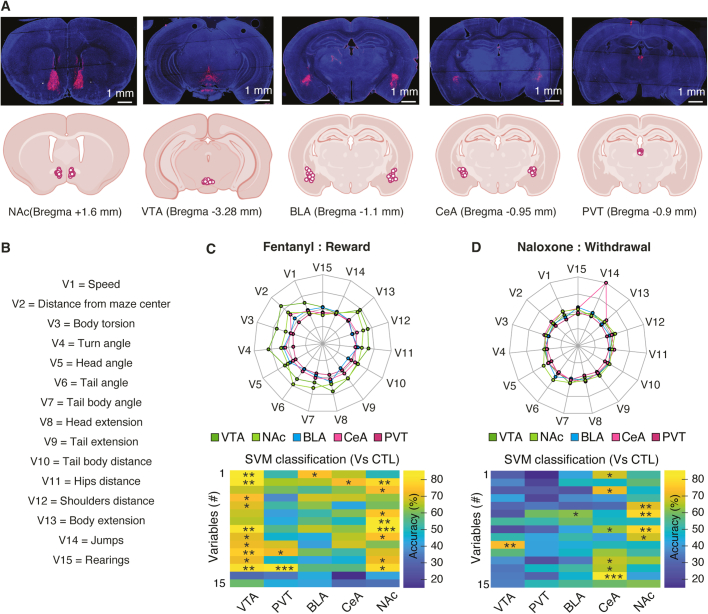
Unbiased behavioral validation of µORs deletion after fentanyl and naloxone treatments. (**A**) Top: Representative example of the injection site in the different brain regions. Bottom: Localization of the injection centre in the different mice (CTL (*n* = 12); VTA (*n* = 13); NAc (*n* = 13); BLA (*n* = 12); CeA (*n* = 14); PVT (*n* = 13)). (**B**) Names of the variable extracted for DLC tracking. (**C**-**D**) Top: Spider plot representing the multidimensional distance between each experimental groups (µORs KD in different brain regions) vs control group (non-KD) of each time-series quantified after precipitation of withdrawal. Bottom: SVM classification of the 15 variables extracted from DLC in the different experimental groups (µORs KD) vs control group after precipitation of withdrawal. Color code used to evaluate the accuracy of prediction of each variables extract. (estimated p value with permutation test; *p < 0.05, **p < 0.01, ***p < 0.001).

To identify the brain regions that are active during withdrawal, we quantified the expression of the immediate early gene cFos, a proxy of neuronal activity, in eight distinct brain areas (Fig. 1d). We observed that fentanyl on its own increased the number of cFOS-positive cells in regions believed to mediate positive reinforcement, such as VTA or nucleus accumbens (NAc). When precipitating withdrawal in fentanyl-dependent mice, CeA stood out as the sole region with a significant increase in cFOS-positive neurons (Fig. 1e). To test for causality, we deleted (knocked down) µORs by injecting an adeno-associated virus for combined expression of Cre and tdTomato (AAV8-cre-tdTomato) in mice carrying µORs flanked by *loxP* sites (*Oprm1*^*fl/fl*^). We targeted five brain regions and tested the mice for withdrawal after 5 days of fentanyl exposure (Fig. 1f,g). We validated the strategy in VTA and CeA and confirmed that expression of *Oprm1* was significantly decreased (Extended Data Fig. 2). We observed that knockdown of µORs in CeA strongly reduced jumps, whereas immobility time remained unchanged (Fig. 1h,i). To validate our behavioural observations, we searched for subtle withdrawal symptoms that might have escaped our initial observation using a markerless pose estimation suite (DeepLabCut^18^). We implemented HCTSA^19^, a machine-learning algorithm, to evaluate the change in 15 unbiased variables extracted from pose estimation during initial fentanyl injections and withdrawal. This approach revealed that knockdown of µORs in VTA and NAc affected acute fentanyl-induced behaviour, confirming their involvement in positive reinforcement. Additionally, we observed that knockdown of µORs in CeA strongly affected naloxone-induced precipitation withdrawal (Extended Data Fig. 3). Collectively, these experiments suggest that the initiation of positive and negative reinforcement starts with µOR-expressing neuronal populations in distinct brain locations.

## Characterization of µOR-expressing neurons in CeA

**Fig. 2:**
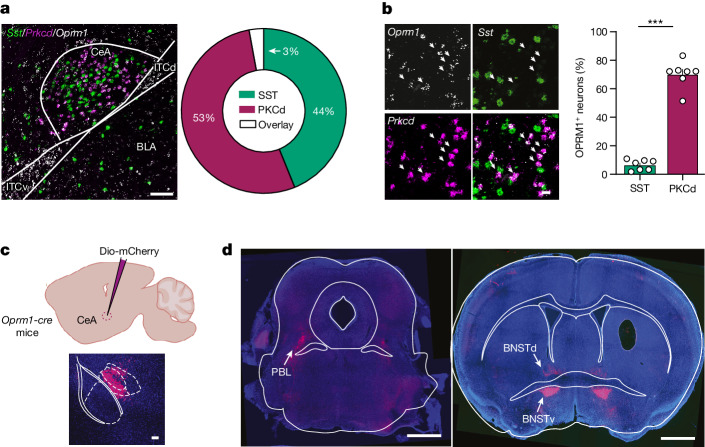
Cellular and anatomical characterization of µOR-expressing CeA neurons. **a**, Left, representative example of CeA imaged by single-molecule fluorescent in situ hybridization (smFISH) for *Oprm1* (blue), somatostatin (*Sst*) (green) and *Prkcd* (red) mRNA. Right, quantification of co-localization between *Sst*- and *Prkcd*-positive neurons (*n* = 7 mice). Scale bar, 100 μm. **b**, Left, representative example of CeA imaged by smFISH for µOR (*Oprm1*) (white), *Sst* (green) and *Prkcd* (red). Right, quantification of *Oprm1*-positive neurons among the *Sst*^+^ and *Prkcd*^+^ populations. Data are mean ± s.e.m. (*n* = 7 mice). Two-sided Mann–Whitney test, ***P = 0.0006. Scale bar, 20 μm. **c**, Schematic of mouse preparation to visualize the µOR-expressing CeA efferent pathway (top) and representative image showing injection (bottom). Scale bar, 100 µm. **d**, Projection site of µOR-expressing CeA neurons in the parabrachial lateral nucleus (PBL) and the ventral bed nucleus of the stria terminalis (BNSTv) and dorsal bed nucleus of the stria terminalis (BNSTd). Scale bars, 1 mm. Source Data

We screened µOR-expressing CeA neurons, looking for co-localization between *Oprm1* transcripts and non-overlapping CeA markers, such as *Sst* and *Prkcd*^20,21^. We found that 69% of *Prkcd* neurons co-expressed *Oprm1*, versus 11% of *Sst* neurons (Fig. 2a,b). We next identified the projections of µOR neurons using a µOR knock-in (*Oprm1-cre*) transgenic mouse line^22^ in which we injected AAV5-hSyn-Dio-mCherry in CeA (Fig. 2c). The two principal target structures were the bed nucleus of the stria terminalis (BNST) and the parabrachial lateral nucleus (Fig. 2d), which have been associated with aversive and pain-related processes, respectively^23–25^.

## Neuronal activity underlying reinforcement

**Fig. 3:**
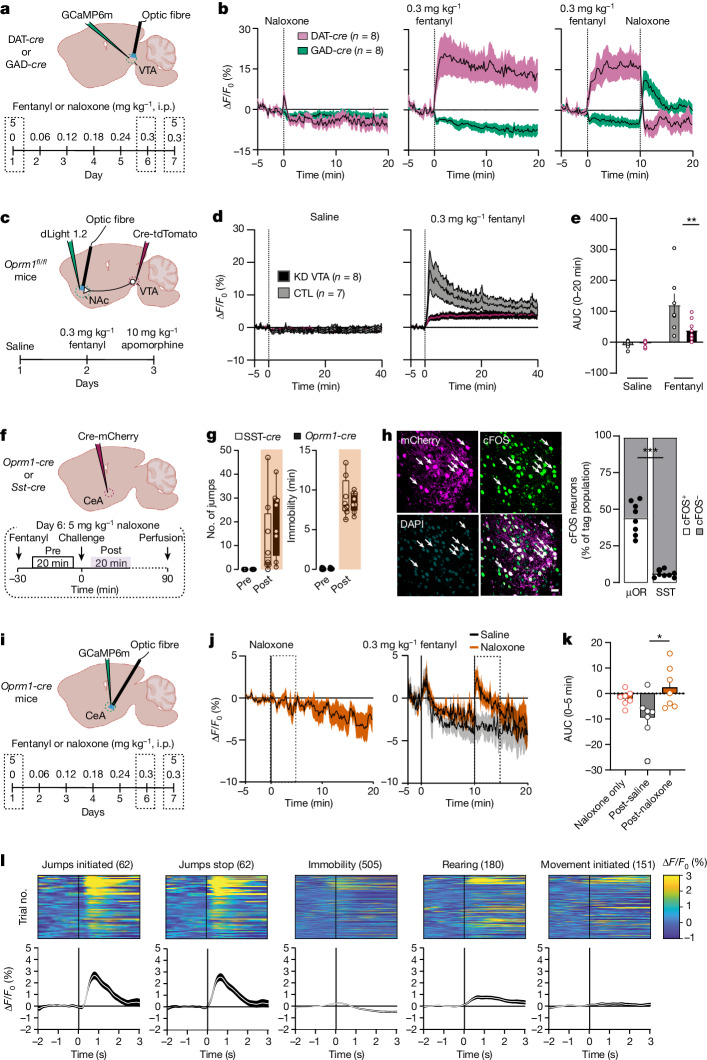
Activity of µOR-expressing neurons during acute and chronic fentanyl exposure. **a**, Top, schematic of mouse preparation for recording Ca^2+^ activity of VTA neurons expressing dopamine and GABA. Bottom, schedule of the recording experiment in fentanyl-dependent mice. Withdrawal is precipitated by naloxone on day 7 (intraperitoneal injection, 5 mg kg^−1^). **b**, Ca^2+^ signal (Δ*F*/*F*_0_) of dopamine (*n* = 8 mice) and GABA (*n* = 8 mice) neurons after intraperitoneal injection of naloxone (5 mg kg^−1^), fentanyl (0.3 mg kg^−1^) and fentanyl plus naloxone. **c**, Top, schematic representation of NAc dLight recordings after µOR deletion in VTA. Bottom, schedule of intraperitoneal injections for dLight recordings after saline, fentanyl (0.3 mg kg^−1^) and apomorphine (10 mg kg^−1^) treatments. **d**, Accumbal dLight signal (Δ*F*/*F*_0_) in mice with deletion of µORs in VTA (*n* = 8 mice) versus control mice (*n* = 7 mice). **e**, Quantification of the area under the curve (AUC) after intraperitoneal injection of saline or fentanyl in mice with deletion of µORs in VTA (*n* = 8 mice) versus control mice (*n* = 7 mice). Two-way repeated measures ANOVA: group effect, *F*_(1,26)_ = 4.371, *P* < 0.05; injection effect, *F*_(1,26)_ = 23.95, *P* < 0.0001; group × injection, *F*_(1,26)_ = 5.777, *P* < 0.05; Bonferroni post hoc analysis, **P = 0.0076. Data are mean ± s.e.m. **f**, Top, schematic of mice preparation to label µOR-expressing neurons in CeA. Bottom, schedule of the experiment to induce fentanyl dependence and precipitation of withdrawal on the challenge day (day 6). **g**, Box plot representation of jumps and immobility time withdrawal symptoms quantified in *Oprm1*-*cre* (*n* = 8 mice) and *Sst*-*cre* (*n* = 8) mice. The centre line is the median, box edges delineate first and third quartiles, and whiskers extend to maximum and minimum values. **h**, Left, representative example of CeA µOR-expressing neurons co-localizing with cFOS (white arrows) after precipitated withdrawal. Right, fraction of cFOS-expressing neurons among CeA µOR or SST-expressing neurons (*n* = 8 mice for SST and *n* = 8 mice for µOR; two-sided unpaired *t*-test, ***P < 0.0001). Scale bar, 20 μm. **i**, Schematic of mouse preparation for Ca^2+^recording of CeA µOR-expressing neurons during precipitation of withdrawal. **j**, Average Ca^2+^ signal (Δ*F*/*F*_0_) of CeA µOR-expressing neurons in naive mice after intraperitoneal injection of naloxone (left) and independent mice after intraperitoneal injection of fentanyl (0.3 mg kg^−1^) plus saline or fentanyl (0.3 mg kg^−1^) plus naloxone (5 mg kg^−1^) (*n* = 7 mice). **k**, Quantification of AUC after intraperitoneal injection of saline or naloxone in naive and dependent mice (*n* = 7 mice; Kruskal–Wallis test for AUC *H*(3) = 7.577, *P* < 0.05; Dunn’s multiple comparisons test, *P = 0.0258). Data are mean ± s.e.m. **l**, Bottom, average Ca^2+^ traces align to different behavioural events during precipitation of withdrawal. Top, trials activity map of each behavioural parameter during precipitation of withdrawal. Source Data

**Extended Data Fig. 4:**
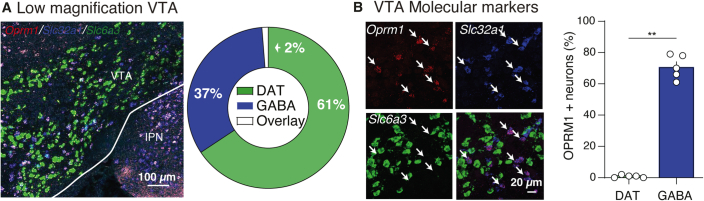
Anatomical validation of VTA µORs expressing neurons. (**A**) Left: Low VTA magnification representative example of fluorescent in situ hybridization for opioid receptor mu 1 (*Oprm1*, Blue), solute carrier family 32 member 1 (*Slc32a1*, green) and solute carrier family 6 member 3 (*Slc6a3*, red) mRNAs (Scale bars: 100 µm). Right: Quantification of colocalization between *Slc32a1* (DAT) and *Slc6a3* (GABA) positive neurons (*n* = 5 mice). (**B**) Left: Fluorescent in situ hybridization representative example of VTA molecular marker for *Oprm1* (µORs, White), *Slc32a1* (GABA, green) and *Slc6a3* (DA, red). Right: Proportion of GABA and DA neurons (Mean ± SEM) within the µORs-expressing neurons (*n* = 5 mice). Mann-Whitney test, two-sided, **p = 0.0079. Source Data

**Extended Data Fig. 5:**
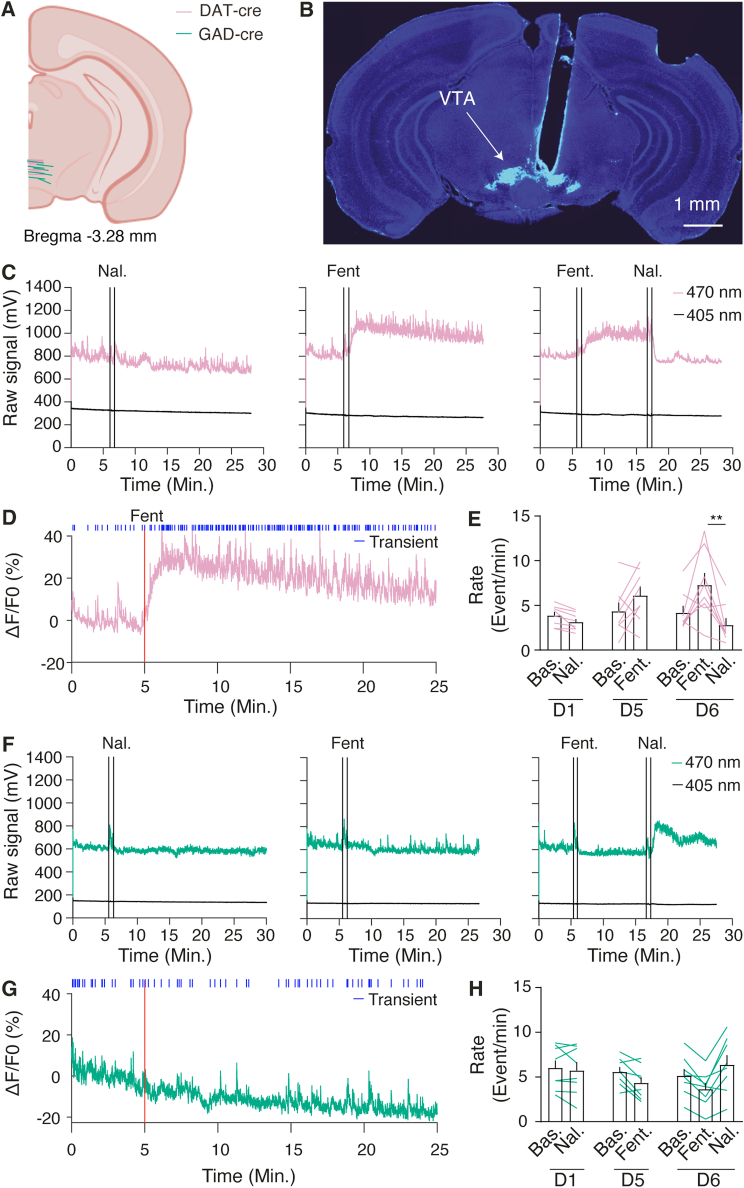
Example traces of evoked DA and GABA activity after pharmacological i.p. injection. (**A**) Localization of the recording site in DAT-cre (*n* = 8) and GAD-cre (*n* = 8) animals. (**B**) Representative histological example of virus infection and fibre implantation in VTA. (**C**,**F**) Raw signal recording example (470 and 405 nm) of GCaMP6m after i.p. injection of Naloxone in naïve mice (Left), after i.p. injection of fentanyl (middle) and after fentanyl plus naloxone i.p. injection (right) in dopaminergic neurons (Pink) and GABAergic neurons (Green). (**D**,**G**) Example Ca^2+^ signal (ΔF/F0) of dopamine (Pink) and GABA (green) VTA neurons and its associated transients identification (Blue). (**E**,**F**) Average transients rate (Mean ± SEM) after i.p. injection of Naloxone in naïve mice, after i.p. injection of fentanyl and after fentanyl plus naloxone i.p. injection in dopaminergic neurons (pink) and GABAergic neurons (green) (For DAT transients, Kruskal-Wallis test H(7) = 21.66, P < 0.01, For GAD transients, Kruskal-Wallis test H(7) = 6.788, P > 0.05, Dunn’s multiple comparisons test, *p < 0.01). Source Data

**Extended Data Fig. 6:**
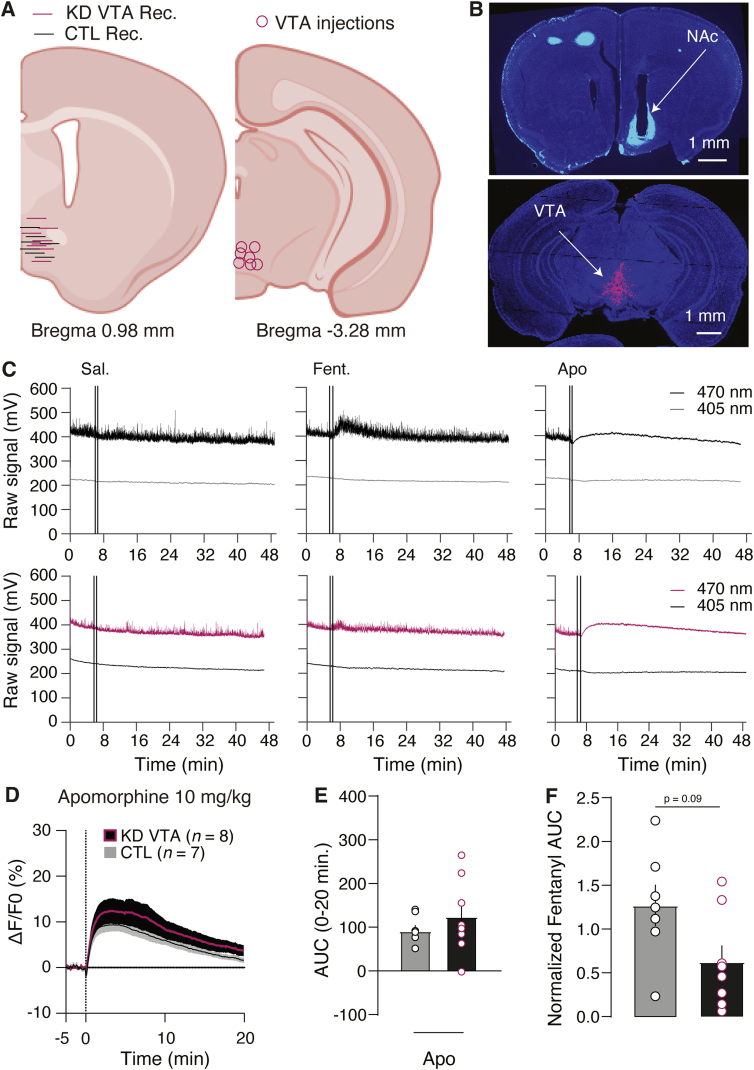
Histological and functional validation of dopamine transients evoked after µORs KD in VTA. (**A**) Localization of the NAc recording site in VTA µORs KD (*n* = 8 mice) and CTL (*n* = 7 mice) animals plus viral infection in VTA. Bottom: (**B**) Representative histological example of fibre implantation in NAc and virus infection in VTA. (**C**) Representative example of NAc raw dLight signal (470 and 405 nm) in control (Top) and VTA µORs KD (Bottom) animals after i.p. injection of saline, Fentanyl (0.3 mg/kg) and apomorphine (10 mg/kg). (**D**) Accumbal dLight signal (ΔF/F0) in VTA µORs KD (red, *n* = 8 mice) vs CTL mice (grey, *n* = 7 mice) after i.p. injection of apomorphine at 10 mg/kg. (**E**) Mean ± SEM of the area under the curve after pharmacological i.p. injection of apomorphine in VTA µORs KD (*n* = 8 mice) and CTL (*n* = 7 mice). (**F**) Normalization of AUC (Mean ± SEM) evoked by fentanyl 0.3 mg/kg i.p. injection compared to the AUC of the partial agonist D1/D2 dLight apomorphine in VTA µORs KD (*n* = 8 mice) and CTL (*n* = 7 mice) (Mann-Whitney test, two-sided, p > 0.05). Source Data

We next monitored the neural activities linked to positive and negative reinforcement using fibre photometry to monitor intracellular Ca^2+^ (using GCaMP6m) during our injection schedule of increasing fentanyl doses (Fig. 3a). Fentanyl inhibited GABA neurons but activated (that is, dis-inhibited) dopamine neurons in VTA; this was confirmed by anatomical localization of µORs in GABA-expressing neurons (Fig. 3a and Extended Data Fig. 4). In fentanyl-naive mice, naloxone had no effect on activity; however, in dependent mice, naloxone injection triggered a rebound of GABA neuronal activity along with transient inhibition of dopamine neurons (Fig. 3b). These activity changes were not associated with a variation of rapid, spontaneous Ca^2+^ transients observed at baseline throughout all recordings (Extended Data Fig. 5). We then monitored dopamine release in the NAc using a genetically encoded dopamine sensor (dLight1.2) after knockdown of µORs in VTA. Fentanyl-evoked transients was reduced by 66% (Fig. 3c–e). To account for individual expression levels, we normalized the dLight recordings to the transients evoked by the partial dopamine D_1_ and D_2_ receptor agonist apomorphine (Extended Data Fig. 6).

We then confirmed that the CeA neurons activated during withdrawal were indeed µOR-expressing cells (Fig. 3f,g). *Oprm1*-*cre* and *Sst*-*cre* mice were injected with AAV5-EF1α-Dio-mCherry to label different CeA populations, followed by cFOS quantification. A large fraction of µOR-expressing CeA neurons was activated after withdrawal precipitation, in stark contrast to the non-overlapping SST population (Fig. 3h). We next monitored the activity of µOR-expressing CeA neurons in vivo with fibre photometry Ca^2+^ imaging (Fig. 3i). In dependent mice, naloxone flipped the activity of µOR-expressing CeA neurons to transient hyperactivity, which was not the case in naive mice (5 mg kg^−1^; Fig. 3j,k). We then correlated this activity with behavioural video during withdrawal and observed an increase of Ca^2+^ signal immediately after jumps and stable neuronal activity during immobility periods (Fig. 3l).

Together, these observations confirm the existence of a disinhibition mechanism involving µORs in VTA responsible for the initiation of positive reinforcement^1,26^. As deletion of µORs in VTA abolished dopamine neuron activity but did not prevent withdrawal, mesolimbic adaptation is unlikely to be the locus for the induction of the negative reinforcement. Conversely, the increased activity of CeA µOR-expressing neurons during withdrawal, time-locked to the end of jumps, indicates that these cells encode an aversive experience.

## Optogenetic positive and negative reinforcement

**Fig. 4:**
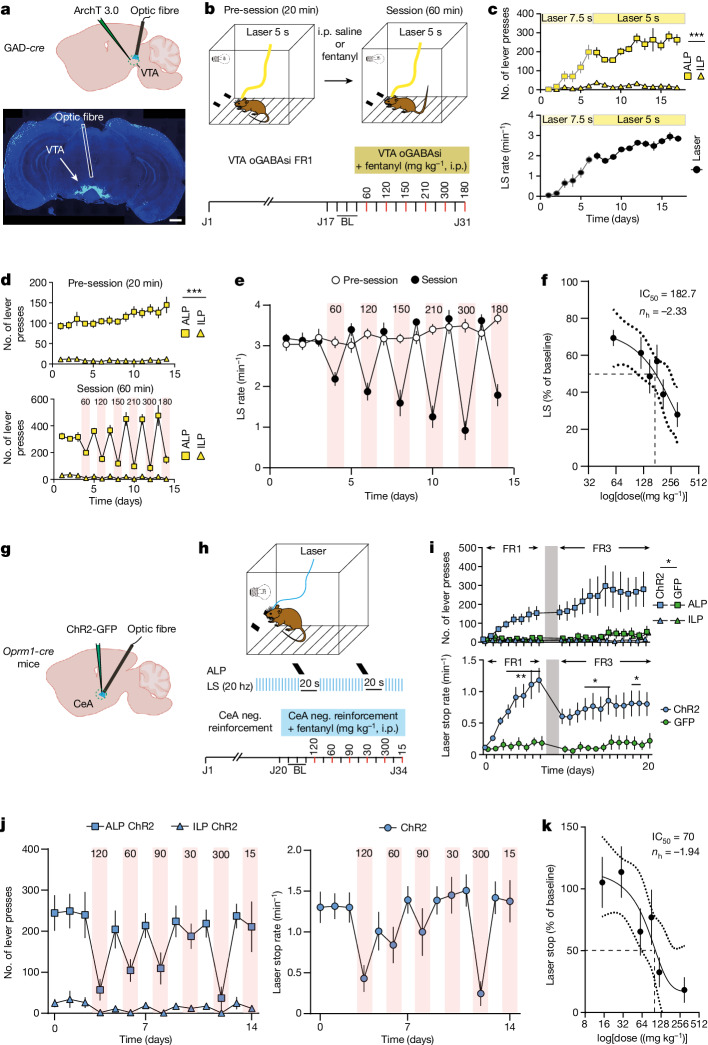
Fentanyl occludes optogenetic mimicry of positive and negative reinforcement. **a**, Schematics for oGABAsi experiments (top) and histological validation (bottom). Scale bar, 1 mm. **b**, Fentanyl occlusion schedule during oGABAsi experiment. **c**, Active lever presses (ALP), inactive lever presses (ILP) and laser stimulations (LS) per minute during operant conditioning (*n* = 9 mice; LP: two-way repeated measures ANOVA, LP effect *F*_(1,8)_ = 203, ***P < 0.001; session effect *F*_(16,128)_ = 12.12, ***P < 0.001; LP × time interaction *F*_(16,128)_ = 11.23, ***P < 0.001). Data are mean ± s.e.m. **d**, ALP and ILP during the pre-session (top) and during the session (bottom) after injection of fentanyl at different doses (*n* = 9 mice; LP pre-session: two-way repeated measures ANOVA, LP effect *F*_(1,8)_ = 198.3, ***P < 0.001; time effect *F*_(13,104)_ = 2.263, *P < 0.05; LP × time interaction *F*_(13,104)_ = 2.586, *P < 0.05) Data are mean ± s.e.m. **e**, Laser stimulations per minute during the pre-session and post-session following intraperitoneal injection of saline or fentanyl at increasing doses (*n* = 9 mice). Data are mean ± s.e.m. **f**, Dose–response curve for fentanyl occlusion of oGABAsi (*n* = 9 mice). Sigmoid fit yielding an IC_50_ of 187.2 μg kg^−1^ and a Hill coefficient of −2.33. Data are mean ± s.e.m. **g**, Schematic of mouse preparation for optogenetic manipulation of CeA µOR-expressing neurons during an operant negative reinforcement task. **h**, Fentanyl occlusion schedule during the operant negative reinforcement task. **i**, ALP and ILP (top) and laser stop (bottom) rates during the FR1 and FR3 operant conditioning phases in control (*n* = 7 mice) or ChR2 (*n* = 8 mice) mice (ALP: two-way repeated measures ANOVA, group effect *F*_(1,13)_ = 6.671, *P < 0.05; time effect *F*_(19,247)_ = 4.64, ***P < 0.001; LP × time interaction *F*_(19,247)_ = 2.59, ***P < 0.001; rate: two-way repeated measures ANOVA, group effect *F*_(1,13)_ = 12.74, *P < 0.05; time effect *F*_(19,247)_ = 6.059, ***P < 0.001; LP × time interaction *F*_(19,247)_ = 3.801, ***P < 0.001). Data are mean ± s.e.m. **j**, ALP and ILP (left) and laser stop (right) after injection of fentanyl at different doses (*n* = 7 mice). Data are mean ± s.e.m. **k**, Dose–response curve for fentanyl occlusion of optogenetically manipulating CeA µOR-expressing neurons in an operant negative reinforcement task (*n* = 7 mice). Sigmoid fit yielding an IC_50_ of 70 μg kg^−1^ and a Hill coefficient of −1.94. Data are mean ± s.e.m. Source Data

**Extended Data Fig. 7:**
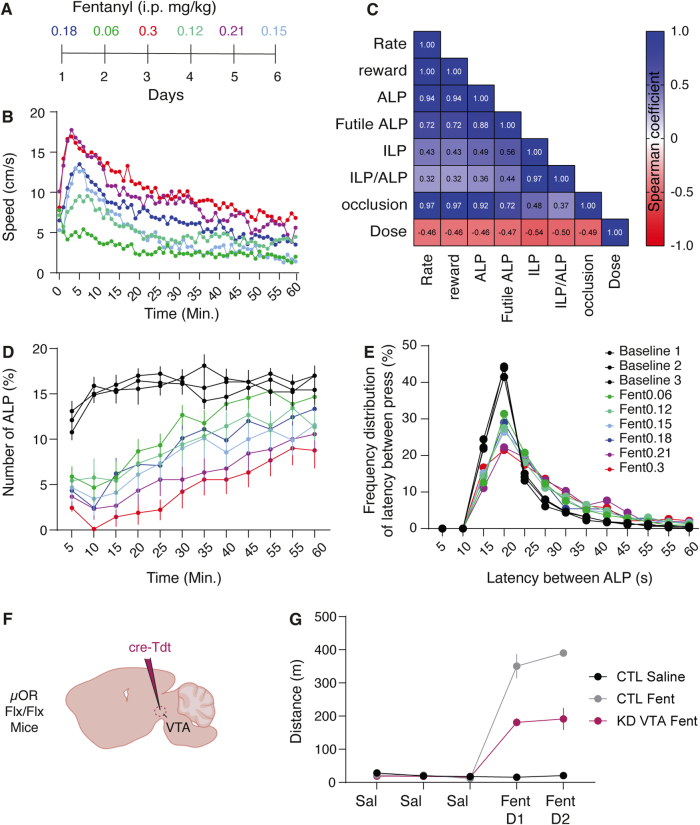
Behavioral effects during oGABAsi-fentanyl occlusion and locomotion reduction after VTA µOR-KD. (**A**) i.p. protocol fentanyl injection. (**B**) Average speed locomotion induced by fentanyl at different doses in 1 h open field test (*n* = 7 mice). (**C**) Correlation matrix of the different behavioral parameters during oGABAsi occlusion. Superimposed are annotated the Spearman correlation coefficient (*n* = 7 mice). (**D**) Active lever press number (ALP, Mean ± SEM) through the session of oGABAsi occlusion after different dose of fentanyl i.p. injected (*n* = 7 mice). (**E**) Frequency distribution of latency between ALP during oGABAsi occlusion experiment after different dose of fentanyl i.p. injected (*n* = 7 mice). (**F**) Schematic of the mice preparation to induce VTA µORs deletion. (**G**) Distance travelled (Mean ± SEM) after i.p. injection of saline (sal) or Fentanyl 0.2 mg/kg in mice with VTA µORs KD (*n* = 4 mice, red), control animal (*n* = 4 mice, grey) or control saline animal (*n* = 4 mice, black).

**Extended Data Fig. 8:**
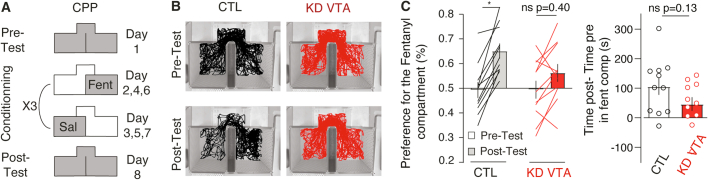
VTA µOR-KD prevents conditioned place preference. (**A**) Schedule of the experiment to induce Place preference with i.p. injection of fentanyl 0.3 mg/kg. (**B**) Representative example of occupancy plot for control mice (Left) and VTA µORs KD mice (Right), before (top) and after (Bottom) conditioning. (**C**) Left: Average preference score (Mean ± SEM) for the fentanyl compartment before and after conditioning in CTL mice (Black, *n* = 11) and VTA µORs KD mice (red, *n* = 10) (Two way RM ANOVA, Session, F_(1,38)_ = 10.91, p < 0.01, Genotype, F_(1,38)_ = 0.8352, p > 0.05, Session x Genotype, F_(16,38)_ = 2.046, p > 0.05; Bonferroni post hoc analysis, **p = 0.029). Right: Average difference of time (in seconds, Mean ± SEM) spent in the fentanyl compartment for CTL mice vs VTA µOR KD mice (Right, Mann-whitney test, two-sided, p > 0.05). Source Data

If VTA dopamine neuron disinhibition is reinforcing, optogenetic self-inhibition of GABA neurons (oGABAsi) should elicit a similar behaviour. To test for this possibility, we infected GAD-*cre* mice with AAV5-EF1α-eArch3.0-Dio-EYFP for expression of the inhibitory opsin ArchT in VTA. The mice then learned to press a lever to turn on an amber laser for oGABAsi on a FR1 schedule (that is, reinforcement is delivered after each response) (5–7.5 s inhibition; Fig. 4a,b). All mice learned this behaviour, reaching a stable rate of three laser inhibitions per minute (Fig. 4c) within the 17 days, confirming the reinforcing nature of the operant behaviour. We then occluded oGABAsi by intraperitoneal injections of fentanyl at increasing doses delivered in a pseudo-random order to reduce the effect of tolerance (Fig. 4d,e). Whereas baseline oGABAsi rates remained constant, fentanyl decreased the performance in a dose-dependent manner with a half-maximal inhibitory concentration (IC_50_) of approximately 180 µg kg^−1^ (Fig. 4f). Although there was a correlation between fentanyl doses, locomotion and lever presses, the mice maintained their pattern of lever pressing. Moreover, when VTA expression of µORs was knocked down, fentanyl-induced locomotion was blunted, suggesting a dual modulation of µOR-expressing VTA neurons in motivation and locomotion behaviour (Extended Data Fig. 7). Thus, optogenetic disinhibition of VTA dopamine neurons is reinforcing and behaviourally occluded with pharmacological disinhibition. We also confirmed the rewarding properties of µORs by blocking CPP via knockdown of µORs in VTA (Extended Data Fig. 8). Together, these data demonstrate that fentanyl exerts its positive reinforcement through a disinhibition mechanism in VTA, causing transient increases of dopamine in NAc.

**Extended Data Fig. 9:**
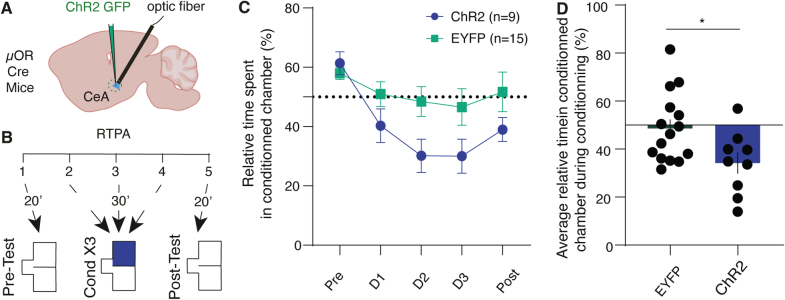
Real-time place aversion (RTPA) induced by CeA µOR-neurons activation. (**A**) Schematic of the mice preparation for optogenetically manipulating CeA µORs-expressing neurons during a real-time place aversion task. (**B**) Schedule of the experiment to induce real-time place aversion with 20 Hz stimulation, consisting of a pre-session (20 min), three sessions of conditioning (30 min) and a post-conditioning session (20 min) (**C**) Relative time spent in the stimulating chamber (Mean ± SEM) during the behavioral task (*n* = 9 for ChR2 group and *n* = 15 for the GFP group). (**D**) Average (Mean ± SEM) relative time spent in the conditioning chamber over the 3 conditioning days (*n* = 9 for ChR2 group and *n* = 15 for the GFP group; Unpaired t test, two-sided, *p = 0.028). Source Data

If µOR-expressing CeA neurons drive negative reinforcement, then tonic optogenetic activation of these neurons should be aversive. We transfected µOR-expressing CeA neurons with an AAV5-EF1α-ChR2(H134R)-Dio-EYFP in *Oprm1*-*cre* mice and continuously stimulated them at 20 Hz. In real-time place aversion (RTPA) trials, mice learned to avoid the stimulated side, indicating that the stimulation was aversive (Extended Data Fig. 9). We then developed an operant task in which mice could press a lever to pause the stimulation for 20 s. The mice quickly learned to stop the aversive stimulation on an FR1 schedule and continued to perform the operant task when switched to an FR3 schedule (that is, three responses are required before reinforcement is delivered) (Fig. 4i). Injection of fentanyl occluded this behaviour in a dose-dependent manner, akin to the oGABAsi experiment (Fig. 4j,k). Together, these data show that µOR-expressing CeA neurons are the cellular triggers for the induction of negative reinforcement.

## Discussion

We demonstrate here that µORs in VTA and CeA neurons induce positive and negative fentanyl reinforcement, respectively. We pinpoint a cellular population responsible for the induction of opioid dependence, an observation distinct from the findings implicating the amygdala in the expression of withdrawal^8^.

For positive reinforcement, µOR-expressing GABA neurons in VTA are the initial target leading to the disinhibition of dopamine neurons. This scenario was proposed after demonstrating the synaptic connectivity of GABA onto dopamine neurons in acute brain slices, but was subsequently challenged on the basis of behavioural, electrophysiological, pharmacological and genetic evidence^27^. For example, dopamine-deficient mice (by targeted deletion of tyrosine hydroxylase and dopamine β-hydroxylase) still exhibit CPP for morphine^28^. However, this was only possible when mice were treated with levodopa and stimulated with caffeine. It is possible that µORs on dopamine D_1_ receptor-expressing medium-sized spiny neurons of the NAc or µOR-expressing glutamate neurons of VTA^29^ also contribute to the reinforcing effects of opioids^30^. VTA GABA projection neurons, which selectively target cholinergic neurons in the NAc^31^, may also be inhibited by fentanyl. This would boost acetylcholine, which can also cause direct dopamine release from axon terminals^32^. As in a previous study using heroin^14^, our data for fentanyl contradict the idea of dopamine-independent reward in naive mice^33^. Finally, although our fibre photometry experiments showed an overall enhanced dopamine neuron activity and accumbal dopamine transients, it remains possible that a subpopulation of dopamine neurons may be inhibited by fentanyl^34,35^. If these neurons code for aversion, their inhibition could contribute to positive reinforcement, but experimental evidence for such a scenario remains elusive.

Our occlusion experiment suggests a circuit convergence between pharmacological and optogenetic disinhibition, with the caveat that a third parameter—fentanyl-induced locomotor activity—could be a potential confound. It remains possible that fentanyl drives the enhanced movements via a distinct circuit, which precludes the mice from touching the lever simply because they are too busy running around. However, since accumbal dopamine signalling contributes to locomotion^36^, an entirely distinct site of action seems unlikely.

The neural mechanisms that underlie negative reinforcement have been conceptualized as an opponent process that builds up with chronic exposure when the subject becomes dependent^2,37^. Brain areas that were initially implicated included the locus coeruleus (LC), which becomes hyperactive during withdrawal, yet locus coeruleus manipulations do not affect withdrawal behaviour^38^. The extended amygdala, comprising CeA, BNST and NAc shell, has also been implicated in the expression of withdrawal symptoms^8,39–41^. CeA neurons may indeed drive the expression of the withdrawal syndrome via an inhibitory projection to VTA^42,43^. These cells express corticotropin releasing-factor (CRF) and are distinct from the µOR-expressing neurons identified in the present study. Moreover, enhanced CRF signalling can result from stress alone and thus would not explain how fentanyl brings about negative reinforcement. The µOR-expressing CeA population largely overlaps with *Prkcd* RNA expression, whereas CeA RNA levels of *Crh* (which encodes CRF) coincide with those of *Sst*^21^, further suggesting distinct populations. Whether the two populations are connected and how this may contribute to opioid withdrawal remains to be investigated. The paraventricular thalamus, parts of the BLA and the BNST (identified as a major target of the µOR-expressing CeA neurons in our study) also convey negative valence^44,45^. In particular, a potentiation of afferents from the PVT to the NAc may contribute to the expression of the withdrawal syndrome downstream of the µOR neurons that we identified. The medial habenula, which expresses a very high density of µORs, may also undergo adaptations that cause dysphoria, perhaps via its projections to the interpeduncular nucleus or to the lateral habenula, an excitatory nucleus that projects to GABA neurons in the tail of VTA (also called rostromedial tegmentum), which can inhibit VTA dopamine neurons^46,47^.

Our use of optogenetic mimicry (oGABAsi) enabled experiments to further the mechanistic understanding of pharmacological actions on identified circuits. This approach may be less suited to investigate physiological phenomena, in which functional diversity within a seemingly homogenous population may be crucial—for example, between a more medial and a more lateral projection from VTA to NAc^48^. Future studies may address whether the difference in the two ascending streams is also relevant in drug addiction.

Although we provide compelling evidence for induction of negative reinforcement in CeA, confirming that withdrawal shares a neural substrate with anxiety, negative reinforcement may not be sufficient to drive self-administration by itself. Negative reinforcement evolves throughout substance use disorder and exerts an additional drive once dependence is established. Our unbiased cFOS screen pointed to µOR-expressing CeA neurons as the locus of induction for negative reinforcement, and future studies may examine how these structures may become downstream targets. Additionally, we demonstrated that our manipulation affects jumps precipitated by withdrawal (a main behavioural symptom in rodent). Nevertheless, we cannot exclude that other brain areas may be involved in the induction or expression of different withdrawal symptoms, as previously suggested^8^. Future studies will aim to identify the site of convergence of the positive and negative reinforcement circuits to promote the transition to compulsion. Such dual drive could explain why fentanyl and opioids in general are more addictive than psychostimulants, for which negative reinforcement is less pronounced.

These findings may also help to refine current addiction management, such as oral substitution with a long-acting opioids. Given once daily, methadone prevents withdrawal but remains reinforcing, which may help improve the quality of life by eliminating negative reinforcement and facilitating the transition to abstinence^49^. The extensions of the current circuit model^18,19,50^ by adding circuits of negative reinforcement is a step towards a comprehensive understanding of addiction. Circuit-specific interventions may enable targeting of positive and negative reinforcement separately to enhance efficacy.

## Methods

### Mice

C57BL/6 J mice were purchased from Charles River. DAT-IRES-*cre* (B6.SJL-*Slc6a3*^*tm1.1(cre)Bkmn*^/J), GAD-IRES-*cre* (*Gad2*^*tm2(cre)Z*^) and µOR *fl*/*fl* (B6;129-*Oprm1*^*tm1.1Cgrf*^/KffJ or *Oprm1*^*fl/fl*^) mice were from the Jackson Laboratory, µOR *cre*/*cre* (B6N-*Oprm1*^*tmT2A-eGFP/cre(ICS)*^/Kf or *Oprm1*-*cre*) mice were provided by B. L. Kieffer and SST-IRES-*cre* (*Sst*^*tm2.1(cre)Zjh*^/J) mice were provided by A. Holtmaat. On arrival, the mice were given a period of 7 days for habituation. Both male and female mice, aged from 8–12 weeks, were used and group housed in a temperature-controlled (21 ± 2 °C) and humidity-controlled environment (50 ± 5%), under a 12 h light/dark cycle, and provided with food and water ad libitum. After surgical procedures, mice were single housed and recovered for at least 7 days before any experimental procedure. Weights and sexes were distributed homogeneously among the groups if possible. All behavioural procedures were performed during the light cycle. All procedures were approved by the Institutional Animal Care and Use Committee of the University of Geneva and by the animal welfare committee of the Canton of Geneva, in accordance with Swiss law.

### Virus injection and implantation

Mice (age 8–12 weeks) were anaesthetized with a mixture of isoflurane (induction 3%, maintenance 1.5%, Attane) and O_2_ (compact anaesthesia station from Minerve) during surgery and then secured in a stereotaxic frame (Stoeling). Before craniotomy, body temperature was maintained at 37 °C with a temperature controller system, and Lacryvisc (Alcon, Switzerland) was applied to prevent eyes from dehydration. For VTA recording of the different neuronal subtypes (anterior posterior (AP): −3.28; medio–lateral (ML): −0.9; dorso-ventral (DV): −4.3; with a 10° angle) or recording of CeA µOR-expressing neurons (AP: −0.9; ML: −2.8; DV: −3.9) mice were injected with an AAV-DJ-EF1α-FLEX-GCaMP6m (respectively 400 and 150 nl) produced at Stanford University vector core. For the recording of dopamine release, an AAV5-CAG-dLight1.2 (400 nl, from Addgene) was unilaterally injected in NAc (AP: +1.5; ML: −0.7; DV: −4.3). For knockdown experiments, µOR *fl/fl* mice were injected with an AAV8-hSyn-cre-tdTomato or the control virus AAV5-hSyn-mCherry (150 to 400 nl) in VTA (AP: −3.28; ML: −0.9; DV: −4.3, with an angle of 10°), NAc (AP: +1,5; ML: −0,7; DV: −4.3), BLA (AP: −1.2; ML: −3.2; DV: −4.2), PVT (AP: −0.9; ML: −0.4; DV: −3, with an angle of 10°) and CeA (AP: −0.9; ML: −2.8; DV: −3.9). For ISH (RNAscope) in wild-type or µOR *fl*/*fl* mice, 250 nl of an AAV8-hSyn-cre was injected in VTA (AP: −3.28; ML: −0.9; DV: −4.3, with an angle of 10°) or CeA (AP: −0.9; ML: −2.8; DV: −3.9). For immunohistochemistry experiments, *Oprm1*-*cre* or *Sst-cre* mice were injected with an AAV5-hSyn-Dio-mCherry in CeA (AP: −0.9; ML: −2.8; DV: −3.9). Finally, for oGABAsi experiments, AAV5-EF1α-eArch3.0-Dio-EYFP was injected in VTA (AP: −3.28; ML: −0.9; DV: −4.3, with an angle of 10°) and for CeA optogenetic manipulation of negative reinforcement an AAV5-EF1α-ChR2(H134R)-Dio-EYFP or the control virus was injected in CeA (AP: −0.9; ML: −2.8; DV: −3.9).

During the same surgical procedure, for in vivo recording of Ca^2+^ and dopamine release, an optic fibre (0.4 mm diameter, MFC_400/430_0.48_4mm_ZF2.5(G)FLT, Doric Lenses) was implanted and same for optogenetic experiment (oGABAsi and negative reinforcement) (0.2 mm diameter, FOC-W-1.25-200-0.37-5.0, Inper). Three screws were fixed into the skull to secure the optical implant, then the optic fibre was lowered 200 µm above the injection site and secure using dental cement. After surgery, mice were allowed to recover for 7 days and were habituated to handling.

### Behavioural apparatus

The behavioural experiment on precipitation of withdrawal (knockdown, cFOS) as well as fibre photometry recording of calcium (Ca^2+^) GCaMP6m took place in a custom build chamber situated in a sound-attenuated chamber (Med Associates). The experiment chambers consist of a white Plexiglas square chamber (20 × 20 × 25 cm) surmounted by a video camera (Cineplex from Plexon) recording at a rate of 40 frames per second. On top of the chamber, a white transparent piece of Plexiglas with a hole at the centre was inserted to prevent mice from escaping. For fibre photometry recording of dopamine release evoked by fentanyl 0.3 mg kg^−1^ and apomorphine 10 mg kg^−1^, the experiment took place in a transparent custom-built open field (30 × 30 × 20 cm) surmounted by a FLIR camera (Blackfly S) recording at 30 Hz. oGABAsi and negative reinforcement experiments took place in an operant chamber (ENV-307A-CT, Med Associates) situated in sound-attenuating cubicle (Med Associates) consisting of a metal/Plexiglas square chamber (15.9 × 14 × 12.7 cm) with a grid floor in which two retractable levers were present on both sides of one wall surmounted by two cues light. The apparatus was controlled and data captured using a PC running MED-PC IV (Med Associates). For CPP or RTPA experiment, a three-compartment chamber (Med Associates) was used. The apparatus consists of two chambers separated by a corridor with equal surface, but distinct walls drawings and floor texture. On top of the context, a FLIR camera recording at 30 Hz (for CPP) or a camera connected to Cineplex (Plexon for RTPA) was used. Finally, for the locomotor response to different intraperitoneal injections, the experiment took place in a transparent custom-built open field (30 × 30 × 20 cm) surmounted by a camera connected to the Cineplex system to track the centre of gravity.

### Behavioural paradigm

#### Dependency and withdrawal precipitation

Mice were first habituated to the intraperitoneal injection of saline at least for 3 consecutive days. Then increasing dose of fentanyl 0.06, 0.12, 0.18, 0.24 and 0.3 mg kg^−1^ (both injections at 10 ml kg^−1^) were injected intraperitoneally in their home cage to create dependency. On the challenge day, mice were injected with a dose of fentanyl at 0.3 mg kg^−1^ and put back in their home cage for 10 min. Then the behaviour was assessed in the video-tracking apparatus for 20 min (pre-period, reward). 30 min after the intraperitoneal injection of fentanyl, naloxone was injected intraperitoneally at a dose of 5 mg kg^−1^ (injection at dose of 10 ml kg^−1^) and the mice put back in the apparatus directly to evaluate precipitation withdrawal symptoms for 20 min (post period, withdrawal). Precipitation of withdrawal was manually scored by quantifying rearings, jumps, body licking, wet-dog shakes and defecations. Furthermore, immobility (2 s of immobility) and distance travelled (in metres) were extracted from the video track.

### Optogenetic experiment

For optogenetic experiments, the implanted optic fibres were connected via patch cords (oGABASI, MFO-F-W1.25-200-0.37-100, negative reinforcement, BFO-1×2-F-W1.25-200-0.37-30, Inper) to a rotary joint (FRJ_1 × 2_FC-2FC; Doric Lenses), suspended above the operant chamber. A second patch cord was connected from the rotary joint to a blue or orange DPSS laser (SDL-473–100 mW, SDL-593–100 mW, respectively; Shanghai Dream Lasers) positioned outside of the context. Laser power was typically 15–20 mW measured at the end of each patch cord. A mechanical shutter was used to control laser output (SR474 driver with SR476 shutter head; Stanford Research Systems, aligned using a connectorized mechanical shutter adapter; Doric Lenses).

oGABAsi experiment (*n* = 9 mice) was designed on a fixed ratio 1 schedule (FR1) consisting of 1 h session daily during the conditioning phase and then two sessions for the occlusion experiment (20 min for pre-session and 1 h for post-session). Each ALP was associated with a cue light of 2 s, and, 5 s later, a continuous laser inhibition of GABA neurons lasting 7.5 s the first 7 days and 5 s the consecutive sessions, to reduce the time of optogenetic inhibition. From the ALP to the end of the optogenetic stimulation, every press on the ALP was recorded but did not initiate a protocol of stimulation (time-out period). The occlusion experiment was realized over 15 days and started by a 20-min pre-session. Then mice were injected intraperitoneally with saline (during baseline and recovery days) or fentanyl at different doses (0.06, 0.12, 0.15, 0.21, 0.3 and 0.18 mg kg^−1^) before the start of the session that lasted 60 min.

The negative reinforcement experiment (*n* = 8 mice for the ChR2 group and *n* = 7 mice for the EYP group, all female) was designed on a 1 h FR1 schedule for 6 days followed by 1 h FR3 schedule for 12 days. The mouse could stop continuous optogenetic stimulation at 20 Hz (5 ms pulse every 50 ms for 1 s every 2 s) by pressing on an ALP. Each ALP was associated with a cue light that lasted 2 s and a pause of the optogenetic stimulation for 20 s. From the ALP to the end of pause of optogenetic stimulation every press on the ALP was recorded but did not initiate a protocol of stimulation pause (time-out period). The occlusion experiment was realized over 12 days consisting of 3 days of baseline followed by 9 days where an injection of fentanyl at different doses was realized every other day (0.12, 0.06, 0.09, 0.3, 0.015 mg kg^−1^). During the baseline or the recovery day, mice were injected intraperitoneally with saline.

### CPP and RTPA

For the CPP experiment (*n* = 10 for the VTA knockdown group and *n* = 11 for CTL group), mice were habituated to saline intraperitoneal injection at least 3 days before the beginning of the behaviour. On day 1 (pre-test), mice were placed in the corridor and allowed to explore both sides of the context for 20 min. Then 6 days of 20 min conditioning were realized by intraperitoneal injection of saline or fentanyl at 0.3 mg kg^−1^ in a randomly assigned side of the context. On the last day, the place preference was assessed by allowing the mouse to freely explore both sides of the context (post-test). Mice were video-tracked, and the time spent in each compartment was calculated offline using a markerless pose estimation method (DLC) and a custom-made Matlab script. The centre of gravity was used to assess the time spent in each of the three compartments (corridor, saline, or fentanyl context). CPP was calculated by computing the time spent in the fentanyl compartment divided by the time spent in both compartments per session.

To achieve real-time place aversion (RTPA), a camera linked to a Cineplex system (Plexon) was used to continuously video-track the mouse within the given context. When the centre of gravity was detected on one side of the context, an uninterrupted digital signal was transmitted to an Arduino device. This digital signal was then conveyed to an Arduino device linked to a blue laser to produce the stimulation pattern utilized in the negative reinforcement task (20 Hz; 5 ms pulse every 50 ms for 1 s every 2 s). After 4–5 weeks of viral expression, mice (*n* = 9 for ChR2 group and *n* = 15 for EYFP group) were habituated for 3 days of experimenter manipulations and to the connection of the cable. On day 1 (pre-test), mice were free to explore for 20 min both sides of the context and we assessed their place preference. On days 2, 3 and 4 mice were free to explore both sides of the context for 30 min. During this phase, when the centre of gravity of the mouse entered the preferred side, a stimulation was sent until the mouse left this side of the context. On day 5 (post-test), mice were free to explore for 20 min both sides of the context where we assessed again their place preference. RTPA was calculated by computing the time spent in the stimulated compartment divided by the time spent in both compartments per session.

### Locomotor response to drug injection

For the fentanyl dose–response on locomotion, mice were first habituated to saline injection for 3 days. Then we randomly daily injected fentanyl (0.06, 0.12, 0.15, 0.18, 0.21 and 0.3 mg kg^−1^) over 6 days and assessed the locomotor response during 1 h. For the locomotor response in VTA µOR-knockdown mice versus controls, we injected saline intraperitoneal for 3 consecutive days, followed by fentanyl (at 0.2 mg kg^−1^) the next 2 days. A control group was used were we injected saline intraperitoneally over 5 days.

### Fibre photometry recordings

After 4–5 weeks of viral expression, mice were first habituated to handling, to the connection cable and intraperitoneal injection of saline for 3 days before testing. On the testing day, mice were connected to the fibre photometry cable and placed in the apparatus for 3 min of habituation before the start of recording. For the study of dopamine release evoked in the VTA knockdown mice vs control, 5 min of baseline fluorescence were made before the intraperitoneal injection, and then the change of fluorescence was monitored during 40 min. Mice were injected intraperitoneally for 3 consecutive days respectively with saline, fentanyl (0.3 mg kg^−1^), apomorphine (10 mg kg^−1^). For the recording of the neuronal activity (CeA and VTA) during opioid dependency and withdrawal, mice were recorded during 5 min of baseline and then 20 min after naloxone alone (5 mg kg^−1^), fentanyl (0.3 mg kg^−1^). To reduce the entangling of the cable on the challenge day, 20 min after the fentanyl intraperitoneal injection, the photoreceiver was stopped and the cable disentangled and switched on 5 min before the intraperitoneal saline or naloxone (5 mg kg^−1^) injection. Finally, the neuronal activity was recorded for 20 min.

Fibre photometry was performed as before, and data were collected with TDT Synapse v.84 (Tucker Davis). During recordings, excitation (470 nm, M470F3, Thorlabs) and control LED light (405 nm, M405FP1, Thorlabs) were passed through excitation filters and focused onto a patch cord. The fibre patch cord was connected to the chronically implanted fibre, and emission light (500–550 nm) was collected through the same fibre and passed onto a photoreceiver (Newport 2151, Doric Lenses). After pre-amplification by the photoreceiver (2 × 10^10 ^V/A) the signal was digitized, demodulated and stored using a signal processor (RZ5P, Tucker Davis Technologies).

The data were analysed using MATLABR2020 (MathWorks). First, the signal during baseline acquisition originating from the 405 nm excitation source was linearly regressed to the signal originating from the 470 nm excitation source, and scaled to the 470 nm originating signal. Δ*F*/*F* was then computed as (470 nm signal – fitted 405 nm signal)/fitted 405 nm signal. Finally, the Δ*F*/*F* was binned into 10-s time bins to plot an average graph, additionally to no binning for the study of transient activity evoked by the intraperitoneal injection. Transients were detected using the Matlab function findpeaks, where peaks were defined as a prominence greater than 2 standard deviations of the Δ*F*/*F* during baseline recording. For the calculation of the area under the curve (AUC), we used the Matlab function trapz Finally, for the normalization of the AUC to apomorphine we computed the ratio of AUC evoked by apomorphine injection to the one evoked by fentanyl injection.

### Histological analysis

Ninety minutes after the precipitation of withdrawal, mice were injected with a lethal dose of pentobarbital (150 mg kg^−1^) and perfused transcardially with PBS and 4% paraformaldehyde solution. Brains were post-fixed overnight at 4 °C. Coronal sections (60 μm) of the region of interest were cut with a vibratome. Immunostaining started by blocking slices in PBS 10% BSA and 0.3% Triton X-100 followed by 48 h incubation in PBS 3% BSA and 0.3%Triton X-100 with primary antibody: rabbit polyclonal anti-cFOS (1:5,000, from SySy, 226003). After three 15 min washes in PBS at room temperature, slices were incubated with 1:500 Alexa-conjugated secondary antibodies against rabbit (Alexa-Fluor 488, Life Technologies, A1108). Then slices were washed three times in PBS. Slices were mounted and covered on microscope slides using DAPI mounting medium vectashield. Images were obtained in a confocal laser-scanning microscopy Leica SP8 confocal microscope using additional 350-nm laser with a 40×/0.7 NA oil immersion. Analysis was performed in at least three sections per mouse per structure of interest. Semi-manual quantification of cFOS was made by an experimenter who was blind to the experimental conditions. For the visualization of dLight expression, after slicing at 60 μm, slices were incubated with a primary antibody (1:500, rabbit polyclonal anti-GFP, Invitrogen, A11122) overnight at 4 °C and the secondary antibody (1:500, Alexa goat anti-rabbit, Life Technologies, A1108) for 2 h at room temperature.

### In situ hybridization

Staining for *Oprm1*, *Slc6a3*, *Slc32a1*, *Sst* and *Prkcd* mRNAs was performed by smFISH. Brains from 7 C57BL/6 J 12-week-old mice were rapidly extracted and snap-frozen on dry ice and stored at −80 °C until use. VTA and CeA coronal sections (14 μm) were collected directly onto Superfrost Plus slides (Fisher Scientific). RNAscope Fluorescent Multiplex labelling kit (ACDBio 323110) was used to perform the smFISH assay according to manufacturer’s recommendations. Probes used for staining are *Oprm1* (ACDBio 315841), *Slc6a3* (ACDBio 315441-C3), *Slc32a1* (ACDBio 319191-C2), *Sst* (ACDBio 404631-C2) and *Prkcd* (ACDBio 441791-C3). After incubation with fluorescently labelled probes, slides were counterstained with DAPI and mounted with ProLong Diamond Antifade mounting medium (Thermo Fisher Scientific P36961). Fluorescence images of labelled cells were captured using sequential laser-scanning confocal microscopy (Leica SP8) and co-localization was quantified manually. For the validation of the VTA or CeA µOR knockdown we automatically counted the number of puncta per slide compared to control condition using ImageJ software.

### Video data analysis

The videos, which have a resolution of 640×480 and a frame rate of 40 fps, were analysed with DeepLabCut^18^. From a subset of videos, we extracted 25 frames per video using the kmeans algorithm to ensure diversity and labelled them manually. The labelling comprised 15 points of interest (four corners of the box, nose, both ears, both shoulders, body centre, both hips, and base, middle and end of the tail). The labelled images were divided into a training set (90%) and a test set (10%) and a model was trained using ResNet-50 and 800,000 iterations. The resulting error was 2.24 pixels on the training set, and 6.01 pixels on the testing set. The model was used to extract the *xy* coordinates of the previously mentioned points of interest throughout the videos. These coordinates were corrected in the following way: the points with low confidence (<0.05) and the outliers in speed or position were replaced by a value obtained by a cubic interpolation. The whole set of coordinates was also smoothed with a moving average filter of width 5.

The body parts coordinates were used to define 14 relative variables, namely the body extension (distance between the middle of the shoulders and the middle of the hips), the distance between the shoulders, the distance between the hips, the distance between the middle of the tail and the body centre, the tail extension (distance between the base and the end of the tail), the head extension (distance between the nose and the middle of the ears), the angle between the body and the tail, the angle in the middle of the tail, the angle between the head and the body, the rotation of the body with respect to a vertical line, the distance between the centre of the mouse body and the centre of the box, the body torsion (ratio of distance between shoulder and hip on the left vs on the right), the speed and rearing.

We defined a 15th variable describing the likelihood of a jump occurring on each frame. For this purpose, we used the fact that the tracking confidence (values between 0 and 1) would drop during jumps because the mouse would leave the frame for a few milliseconds. Knowing that the tracking confidence was close to perfect while the mouse was in frame, the probability of a jump happening can be roughly approximated by *P*(jump) ≈ 1 − (tracking confidence). Pairing this observation with a condition on a big enough speed preceding the loss of tracking allows a refinement of the detection of jumps, as we avoid classifying bad tracking as a jump. More precisely, a sequence of consecutive frames was considered as a jump if the confidence of tracking went below the fixed threshold of 50% and the speed around the loss of tracking went above the fixed threshold of 10 cm s^−1^. These thresholds were defined for the automatic jump detection to closely match the jumps observed during careful examination of a few videos.

For each mouse, we thereby obtain 15 time series (one per variable). The goal is to compare them and see if there are differences between the control group and each one of the experimental groups. Since a direct comparison between time series is not possible, we use hctsa^19^ to perform feature extraction: it evaluates more than 7,000 operations on each time series. A given time series is hence characterized by a vector with more than 7,000 entries containing the evaluated operations. For each of the 15 variables, we assess the similarity between a certain experimental group of mice (knockdown of µORs in different brain regions) and the control group (non-knockdown) by training a linear SVM classifier with 5 repeats of 5 folds cross validation on the characterizing vectors. We compute the mean balanced accuracy: mean balanced accuracy = (sensitivity + specificity)/2. The significance of the results is obtained by comparing our original accuracy with 1,000 repeats of a classification on shuffled data.

### Statistical analysis and reproducibility

Data were analysed with Microsoft excel 16.16.05 and GraphPad prism 10.0.2. Sample size were estimated with G*power (HHU). For each experiment, a minimum of two replications were conducted by experimenters. Statistical analysis was performed in GraphPad Prism 9. For all tests, the significance threshold was placed at *α* = 0.05. Gaussian distribution was evaluated using the D’Agostino and Pearson normality test. Multiple comparisons were first subject to mixed-factor ANOVA or Kruskal–Wallis test (defining both between- and/or within-group factors), respectively, for normally distributed and non-normally distributed data. Where significant main effects or interactions between factors were found (*P* < 0.05), further comparisons were made for normally distributed data by a two-tailed Student’s *t*-test with Bonferonni corrections applied when appropriate or a Dunn test for non-normally distributed data (that is, the level of significance equalled 0.05 divided by the number of comparisons). Mann–Whitney or Wilcoxon tests were used for non-Gaussian distributions when appropriate. For normally distributed data, single comparisons of between- or within-group measures were made by two-tailed unpaired or paired Student’s *t*-test, respectively.

### Reporting summary

Further information on research design is available in the Nature Portfolio Reporting Summary linked to this article.

## Online content

Any methods, additional references, Nature Portfolio reporting summaries, source data, extended data, supplementary information, acknowledgements, peer review information; details of author contributions and competing interests; and statements of data and code availability are available at 10.1038/s41586-024-07440-x.

### Supplementary information

Reporting Summary

### Source data

Source Data Fig. 1–4 and Source Data Extended Data Fig. 1, 2, 4, 5, 6, 8, 9

## Data Availability

The datasets generated during and/or analysed during the current study are available in the Zenodo repository at 10.5281/zenodo.10890957 (ref. ^51^). Source data are provided with this paper.

## Code availability

The Matlab code used to analyse the raw fibre photometry data is provided in the Zenodo repository at 10.5281/zenodo.10890957 (ref. ^51^).
