# Supplementary material for: Distinct µ-opioid ensembles trigger positive and negative fentanyl reinforcement
Source: Nature. 2024 May 22;630(8015):141–8. doi: 10.1038/s41586-024-07440-x (PMC11153127; doi:10.1038/s41586-024-07440-x)
Supplement: Supplementary file 1 — Reporting Summary [file 41586_2024_7440_MOESM1_ESM.pdf]

nature portfolio

Corresponding author(s): Prof. Christian Lüscher

Last updated by author(s): Apr 2, 2024

Reporting Summary

Nature Portfolio wishes to improve the reproducibility of the work that we publish. This form provides structure for consistency and transparency in reporting. For further information on Nature Portfolio policies, see our Editorial Policies and the Editorial Policy Checklist.

Statistics

For all statistical analyses, confirm that the following items are present in the figure legend, table legend, main text, or Methods section.

n/a	Confirmed	
☐	☒	The exact sample size (*n*) for each experimental group/condition, given as a discrete number and unit of measurement
☐	☒	A statement on whether measurements were taken from distinct samples or whether the same sample was measured repeatedly
☐	☒	The statistical test(s) used AND whether they are one- or two-sided<br>*Only common tests should be described solely by name; describe more complex techniques in the Methods section.*
☐	☒	A description of all covariates tested
☐	☒	A description of any assumptions or corrections, such as tests of normality and adjustment for multiple comparisons
☐	☒	A full description of the statistical parameters including central tendency (e.g. means) or other basic estimates (e.g. regression coefficient) AND variation (e.g. standard deviation) or associated estimates of uncertainty (e.g. confidence intervals)
☐	☒	For null hypothesis testing, the test statistic (e.g. F, t, r) with confidence intervals, effect sizes, degrees of freedom and P value noted<br>*Give P values as exact values whenever suitable.*
☒	☐	For Bayesian analysis, information on the choice of priors and Markov chain Monte Carlo settings
☒	☐	For hierarchical and complex designs, identification of the appropriate level for tests and full reporting of outcomes
☐	☒	Estimates of effect sizes (e.g. Cohen's *d*, Pearson's *r*), indicating how they were calculated

Our web collection on statistics for biologists contains articles on many of the points above.

Software and code

Policy information about availability of computer code

Data collection	MED-PC IV 4.34 (Operant Behaviour), TDT synapse version 84 Tucker-Davis (Photometry)
Data analysis	MS-Excel 16 16.0.5, GraphPad Prism 10.0.2, Matlab R2019b, ImageJ 1.52n.

For manuscripts utilizing custom algorithms or software that are central to the research but not yet described in published literature, software must be made available to editors and reviewers. We strongly encourage code deposition in a community repository (e.g. GitHub). See the Nature Portfolio guidelines for submitting code & software for further information.

Data

Policy information about availability of data

All manuscripts must include a data availability statement. This statement should provide the following information, where applicable:

- Accession codes, unique identifiers, or web links for publicly available datasets
- A description of any restrictions on data availability
- For clinical datasets or third party data, please ensure that the statement adheres to our policy

Upon publication the raw data and custom matlab code will be available at zenodo (10.5281/zenodo.10890957)

Research involving human participants, their data, or biological material

Policy information about studies with human participants or human data. See also policy information about sex, gender (identity/presentation), and sexual orientation and race, ethnicity and racism.

Reporting on sex and gender	Not applicable
Reporting on race, ethnicity, or other socially relevant groupings	Not applicable
Population characteristics	Not applicable
Recruitment	Not applicable
Ethics oversight	Not applicable

Note that full information on the approval of the study protocol must also be provided in the manuscript.

Field-specific reporting

Please select the one below that is the best fit for your research. If you are not sure, read the appropriate sections before making your selection.

☒ Life sciences ☐ Behavioural & social sciences ☐ Ecological, evolutionary & environmental sciences

For a reference copy of the document with all sections, see nature.com/documents/nr-reporting-summary-flat.pdf

Life sciences study design

All studies must disclose on these points even when the disclosure is negative.

Sample size	Sample size were estimated with a power calculation software (G*Power)
Data exclusions	For fiberphotometry, conditional knock-down and optogenetic : animals where injection and/or fiber implantation were not correct
Replication	A details experimental protocol is provided to facilitate replication by others. Behavior, fiberphotometry, staining and genetic experiment were replicated in at least 2 or more cohort, by two experimentater when possible.
Randomization	Mice were randomly assigned to treatments condition. Treatment condition were randomized for occlusion experiment.
Blinding	Cell counting (c-Fos) was conducted by an experimenter blind to treatment. Fiber photometry and behavior experiments were realized analyzed blind if applicable, but the experimentalist are always aware of the conditions. In situ hybridization for validation of th KD were realized blindly.

Reporting for specific materials, systems and methods

We require information from authors about some types of materials, experimental systems and methods used in many studies. Here, indicate whether each material, system or method listed is relevant to your study. If you are not sure if a list item applies to your research, read the appropriate section before selecting a response.

Materials & experimental systems

n/a	Involved in the study	
☐	☒	Antibodies
☒	☐	Eukaryotic cell lines
☒	☐	Palaeontology and archaeology
☐	☒	Animals and other organisms
☒	☐	Clinical data
☒	☐	Dual use research of concern
☒	☐	Plants

Methods

n/a	Involved in the study	
☒	☐	ChIP-seq
☒	☐	Flow cytometry
☒	☐	MRI-based neuroimaging

Antibodies

Antibodies used	Rabbit polyclonal anti c-Fos synaoptic system 226003; Goat anti-rabbit IgG alexa 488 invitrogen A1108, rabbit polyclonal anti-GFP

Antibodies used	invitrogen A11122
Validation	All primary antibody were suitable for IHC according to the vendor. Anti c-Fos antibody show perfect results in immunostaining experiment like ICC, IHC and IHC-P according to the manufacturer's website. Furthermore, it has been validated in more than 15 publications based on the manifacturer website. Anti-GFP antibody stained selectively in virus infected area in our hand, and was validated more than a 1000 time according to the manufacturer's website.

Animals and other research organisms

Policy information about studies involving animals; ARRIVE guidelines recommended for reporting animal research, and Sex and Gender in Research

Laboratory animals	C57BL/6J, DAT-IRES-Cre (B6.SJL-Slc6a3tm1.1(cre)Bkmn/J), GAD-Cre (Gad2tm2(cre)Z), µOR fl/fl (B6;129-Oprm1tm1.1Cgrf/KffJ), µOR cre/cre (B6N-Oprm1tmT2A-eGFP/Cre(ICS)/Kf) and SST-IRES-cre (SSTtm2.1(cre)Zjh/J) mice lines were used. For all strains, mice ages 10-22 week were used for the experiments.
Wild animals	No wild animals were used in the study
Reporting on sex	For all strain, both male and female mice were use, but if not possible it was stated in the materials and methods.
Field-collected samples	No fields collected samples were used in the study.
Ethics oversight	All procedures were approved by the institutional animal Care and use commitee of the university of Geneva and by the animal welfare committe of the Canton of Geneva, in accordance with the swiss law

Note that full information on the approval of the study protocol must also be provided in the manuscript.
